# A Simulation Framework for the Integration of Artificial Olfaction into Multi-Sensor Mobile Robots

**DOI:** 10.3390/s21062041

**Published:** 2021-03-14

**Authors:** Pepe Ojeda, Javier Monroy, Javier Gonzalez-Jimenez

**Affiliations:** Machine Perception and Intelligent Robotics Group, System Engineering and Automation Department, Biomedical Research Institute of Málaga (IBIMA), Campus de Teatinos, University of Málaga, 29071 Málaga, Spain; jgmonroy@uma.es (J.M.); javiergonzalez@uma.es (J.G.-J.)

**Keywords:** artificial olfaction, computer vision, simulation engine, mobile robotics, CFD, gas dispersion

## Abstract

The simulation of how a gas disperses in a environment is a necessary asset for the development of olfaction-based autonomous agents. A variety of simulators already exist for this purpose, but none of them allows for a sufficiently convenient integration with other types of sensing (such as vision), which hinders the development of advanced, multi-sensor olfactory robotics applications. In this work, we present a framework for the simulation of gas dispersal and sensing alongside vision by integrating GADEN, a state-of-the-art Gas Dispersion Simulator, with the Unity 3D, a video game development engine that is used in many different areas of research and helps with the creation of visually realistic, complex environments. We discuss the motivation for the development of this tool, describe its characteristics, and present some potential use cases that are based on cutting-edge research in the field of olfactory robotics.

## 1. Introduction

Gas-sensing technology offers a powerful tool for environmental monitoring. Numerous problems are caused by uncontrolled or illicit emissions of polluting gases that contribute to global warming [1] or even cause direct harm to human health [2]. Due to the abundance of sources of pollution in many areas (such as combustion-based vehicles, landfills, or fossil fuel power plants), many governments around the world have, in recent times, invested in implementing systems for the measurement and control of such emissions, including large-scale sensor networks that monitor air quality in big cities [3].

The detection and location of accidental emissions is also an important task that needs to be addressed. The detection and identification of these released volatiles is a challenging task. As well as developing better sensors, research on how to use current sensing technology in ways that overcome its limitations has received significant interest in recent years [4,5,6]. Fugitive gas leaks are common and can have devastating effects, not only because of the pollution that they cause, but because of their high flammability being a potential explosive hazard [7]. Even smaller-scale indoors gas emissions can have pernicious effects [8] and must be promptly detected and controlled. These problems have also been tackled through olfaction-based approaches, both in the form of fixed sensor networks [9] and mobile robotic agents [10,11].

Despite the usefulness of such approaches, the development of techniques (commonly referred to as gas distribution mapping [12,13] or gas source localization [14]) to tackle these issues is a challenging task. Because of the limitations of the characteristics of the sensors themselves (such as long response times [15]) and the complexity of gas dispersion phenomena under turbulent airflow conditions, extensive experimentation is often required to design and refine these techniques, as well as to validate any proposed solution [16]. However, carrying out these experiments is far from trivial, as there are many environmental variables (temperature, humidity, wind) that may escape the control of researchers, hindering the reproducibility of experiments. Furthermore, there is no way to obtain a reliable ground-truth of the gas dispersion, which greatly complicates the analysis of any results obtained. For these reasons, the use of simulation tools (often referred to as Gas Dispersion Simulators or *GDS*) is of great aid.

Such simulators already exist, with a wide variety of focuses (e.g., some GDS are designed specifically for robotics applications [17]) and different compromises between the fidelity and computational complexity of the dispersion models employed [18]. However, as the development of olfaction-based techniques advances and evolves, new requirements arise for these tools. A prime example of this is how, in recent years, research into solutions that combine olfaction with other types of sensing (mostly vision) has begun to be carried out [19,20,21,22]. Thus, the development of simulation tools that allow for a proper integration of different sensing systems becomes necessary.

**Figure 1:**
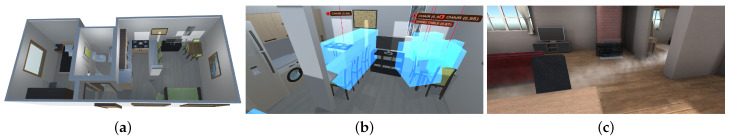
Illustration of the integration of artificial vision and gas sensing systems in a realistic simulated environment. (**a**) Recreation of a small flat including a wide variety of objects. (**b**) Simulated image captured by a virtual camera, where objects have been detected and localized (blue bounding boxes). (**c**) Visualization of a gas plume within a similar environment.

In this work, we present our proposal for integrating gas dispersion in a more sophisticated and graphically realistic simulation framework, offering the integration of visual and olfactory sensing (see Figure 1). We rely on GADEN [17] to perform the gas dispersion simulation and on the Unity Engine [23] to render the visuals. These tools have been chosen for their ability to accurately reflect the complexity of real environments and their ease of integration with other functionalities such as robotic control, path-finding, machine learning, etc.

Our contribution comprises:

- A project in Unity that reads and processes the results of GADEN simulations, making them available to other Unity software.
- The implementation of several models of simulated gas and wind sensors that provide a realistic interface with these data through noisy measurements.
- Three separate models to render the visual representation of a gas plume, according to the desired compromise between computational complexity and visual realism.
- Additional format conversion utilities to facilitate the creation of new simulated environments from pre-existing Unity assets.

We provide a description of the integration of both simulations into one framework and the process of creating a new simulation, as well as examples of use cases that show how it can be applied to improve existing techniques. All the source code of the implementation is made available in a public online repository (https://github.com/MAPIRlab/GADEN_Unity (accessed on 13 March 2021)).

## 2. Related Work

In this section, we will briefly discuss the characteristics and functionalities of GADEN and the Unity Engine, as well as some state-of-the-art alternatives that could be considered for their respective parts of the simulation.

### 2.1. GADEN

GADEN offers the ability to accurately simulate the dispersion of gases in realistic three-dimensional environments by using computational fluid dynamics (*CFD*) to simulate the airflow (e.g., OpenFoam [24]) and employing the filament model to disperse the desired volatiles. Implemented with the tools of the robotic operating system (*ROS*), GADEN is mainly designed for the development of robotics applications, though not limited to them (i.e., it can be used with networks of simulated gas sensors). The environment topology is defined through CAD models, serving for a good degree of detail and enabling an easy setup of new testing scenarios.

**Figure 2:**
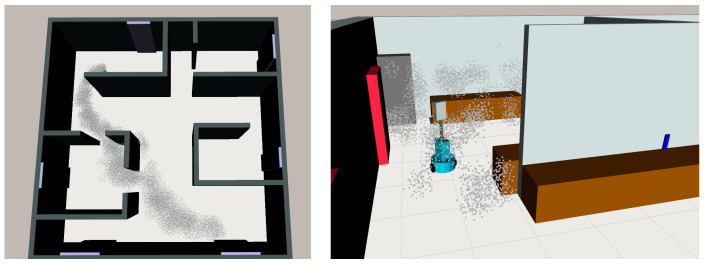
Currently, the results of GADEN simulations have only been integrated for visualization with Rviz. Despite its clarity for user feedback, this visualization does not allow for a high degree of realism and is not suitable for testing image-based algorithms.

Despite its ROS implementation, GADEN itself only handles the computation of the gas dispersion simulation, not the robotic control, the visualization of the gases (e.g., the plume), or other types of sensing such as virtual cameras. Yet, it does offer external tools to visualize the environment and the obtained gas dispersion results through Rviz (see Figure 2); however, this is not a realistic visual representation of the simulated environment, but merely a visual aid for the user. The latter implies that GADEN alone cannot be used to integrate other simulated sensing modalities, such as cameras, hindering its applicability as a simulation test bench when designing advanced, sensor-fusion based alternatives.

Other notable options for gas dispersion simulation include PlumeSim [25], developed for the Player/Stage framework [26], and pure CFD tools such as OpenFOAM [24] or ANSYS [27]. PlumeSim also offers a straightforward integration of the gas simulations with the deployment and control of autonomous agents, but it is limited in terms of the complexity of the environments that can be simulated—e.g., the plume models that can be simulated in it do not take obstacles into account. On the other hand, CFD tools allow for a great deal of precision in the simulation of the gas dispersion, but at a high computational cost, requiring a certain level of CFD expertise from the user and lacking the ability to easily integrate the results of the simulation with other software, such as robotics platforms (see Table 1).

### 2.2. Unity Engine

The Unity engine is a software designed for the development of video games, although its use for other purposes (including architecture, robotics, animation, artificial intelligence, etc.) has been reported [28,29,30,31,32,33]. Because of being designed for video game development, the engine offers powerful tools for creating complex, interactive environments that may be modified at run-time, as well as handling the underlying physics and collisions involved in the simulation of moving objects. Because of the large number of projects that have been developed with the Unity engine, there is also a wide variety of ready-made assets (such as buildings, furniture, etc.) that can be used to quickly set up a new simulated environment with a high degree of visual fidelity.

Furthermore, the engine also provides numerous tools to implement the behaviour of autonomous or controllable agents (such as path-planning, exchange of messages, etc.), allowing the development and testing of algorithms in-platform.

Other similar tools that may be considered as an alternative to the Unity engine exist, including robotics-oriented simulators such as Gazebo [34] and other video game development tools, such as Unreal Engine [35]. Gazebo is available as a ROS meta-package (https://wiki.ros.org/gazebo_ros_pkgs (accessed on 13 March 2021)), which greatly simplifies its integration with GADEN; however, we believe that game engines are a more interesting option for our purposes because of the greater degree of visual realism and computational optimization that they offer and because of their promising future with continuous improvements and notable results.

## 3. Simulation Framework

**Figure 3:**
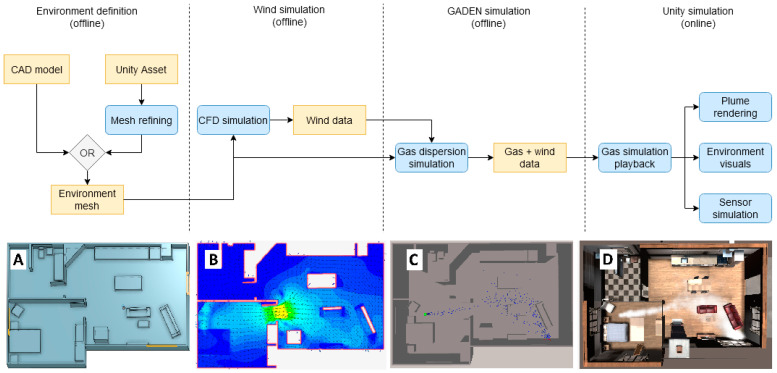
Flow diagram that shows the different steps of the simulation. Most of the work is conducted offline to keep the computational load of the online phase low. (**A**) shows the mesh model that defines the environment, (**B**) shows the airflow vector field computed by CFD, (**C**) shows the filament dispersion simulation in GADEN, and (**D**) shows the integration and visualization of the result in Unity.

Our proposed framework divides the simulation into several phases: defining the environment, simulating the airflow with an external CFD tool, simulating and recording the gas dispersion with GADEN, and integrating the results of the gas dispersion with visual sensing in Unity (Figure 3).

### 3.1. Environment Definition and Airflow Simulation

In order to perform a gas dispersion simulation, GADEN requires as input data both a mesh that defines the environment and a pre-computed 3D map of the airflow (wind vectors). Once an appropriate mesh has been generated, there exist many CFD tools—for example, Simscale [36]—that can be used to generate the needed airflow data.

The mesh that defines the environment in which the simulation is to take place can be generated in several ways (see Figure 3A). This model may be generated with CAD software (as per the traditional workflow of GADEN simulations), or exported from Unity assets. This last option allows for a much more convenient definition of complex, detailed environments, although it must be taken into account that models generated from pre-existing assets do not always conform to the standards of quality required by CFD simulation.

To solve any such problems with exported meshes (e.g., self-intersecting meshes, slit facets), we include in the source code a program that simplifies the model, decreasing the resolution but fixing any defects that impede its use for CFD. A short tutorial on how to achieve similar results with other state-of-the-art mesh manipulation tools is also included, should it become necessary.

### 3.2. Gas Dispersion Simulation

The dispersion of the gas through the environment is simulated in GADEN. The mesh model generated for the CFD simulation is also used in this step to define the topology of the environment, and the simulated airflow data are imported to guide the movements of the gas filaments. The simulation can be configured with a number of parameters, including the location of the gas source (or sources), the types of gas that these sources emit, the rate of emission, etc. The simulation uses the filament model [37], where each “puff” of gas is modelled as a normal distribution of gas concentration around the center point of the filament and the location and size of these puffs is modified over time by diffusion, advection, and buoyancy. The simulated airflow obtained in the previous step is the main factor determining this movement, although turbulence on a scale smaller than the cell size itself can be approximated by adding noise to the movement of the gas filaments. For more details, see [17].

The result of this step is a number of log files that register the gas concentration and wind vectors in each region of the 3D environment for every time step. Both the spatial resolution and the length of the time step employed can be configured by the user, so that an appropriate compromise between precision, computational complexity, and memory usage can be achieved.

### 3.3. Graphics and Sensing Simulation

**Figure 4:**
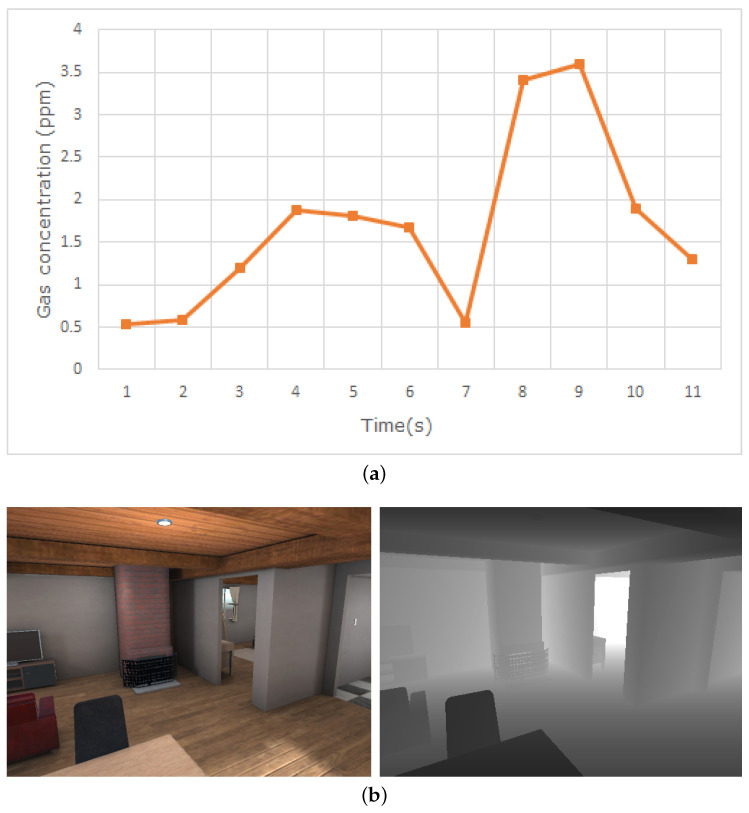
(**a**) Measurements obtained with a simulated photoionization detector at a given point over time. (**b**) RGB and depth images obtained from Unity’s simulated camera.

Lastly, the results of the gas simulation are imported into the Unity engine, where any on-line parts of the simulation are to take place. The Unity Engine handles all the graphical elements of the environment, such as texturing, lighting, etc., which are rendered on GPU. A custom script, also available in the online repository, reads and processes the gas simulation data, making them accessible for any other Unity script that requires them. For this purpose, we provide different models of simulated gas and wind sensors that retrieve these data (Figure 4a).

Two main types of gas sensors are included in the simulation: metal oxide sensors (MOX) and photoionization detectors (PID). Five different models of MOX sensors are included, as well as their response to the presence of gas modelled according to the specifications of the manufacturer and simulating the rise and decay of the measured gas concentration over time. Similarly, the PID sensors are emulated by using the technical specification of the manufacturer of the simulated model to estimate the “weight” of each gas type to the final measured concentration. For more details on the implementation, see the original GADEN publication [17].

Visual sensing is easily simulated through Unity’s camera objects, which can be placed on fixed locations or attached to a moving GameObject and provide an image of the environment as observed from the location of the camera. These images can be accessed through code, either for on-line processing or for storage and later analysis. Unity offers ways to access both the RGB image captured by the camera and a depth image that represents the distance from the camera to the nearest object for every pixel, which can be used to simulate an RGB-D camera (Figure 4b).

**Figure 5:**
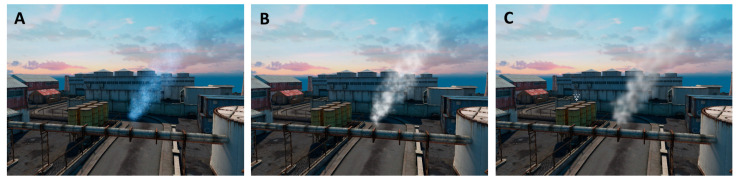
The same gas plume, as visualized with each of the presented methods: (**A**) billboarded 2D assets, (**B**) procedural textures from OpenSimplex noise, and (**C**) volumetric rendering through ray marching.

The visuals of the gas plume can be rendered in several ways (Figure 5), depending on the desired compromise between visual fidelity and computational complexity. Methods that rely on analyzing the shape and movement of the plume itself (i.e., plume imaging; see Section 4.2) will require a higher degree of realism, while in other cases the rendering of the plume only serves as a visual reference for the user.

**Figure 6:**
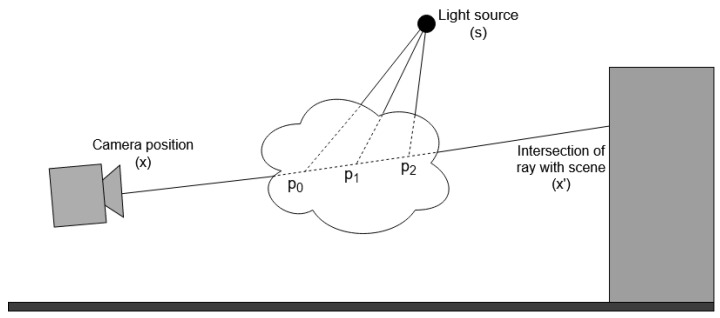
The volumetric rendering of the gas plume is based on taking concentration samples at several points along the view ray to compute the optical depth, and with it the transmittance of the gas puff. Additional rays are cast towards the light source at each sampling point to simulate the scattering of light towards the camera.

- The simplest method is to use Unity’s Particle System objects to represent the gas filaments with billboarded (i.e., always facing towards the camera) pre-existing 2D assets. The user can choose several parameters to configure this effect (color, opacity, etc.). An important feature we introduce in this implementation is the ability to control the visibility of the gas as a function of the existing concentration (gas may only be observable above a certain concentration threshold, or not at all).
  This method requires little work from the user, and represents a significant improvement from previous versions of GADEN, but is not sophisticated enough to achieve a realistic visualization of the gas plume. Therefore, it is appropriate for providing the user a way to visualize the data of the simulation, but should not be employed for plume imaging methods.
- Another option, still based on Unity’s Particle Systems, is to use procedurally generated textures to represent the gas puffs. This allows the asset to change over time, and be modified as the parameters of the filaments change. In practice, since each gas filament is modelled as a gaussian distribution of gas particles, the texture will have that same shape, but be corrupted with some type of coherent noise—the implementation we include in the project uses OpenSimplex noise [38]—to model low-scale turbulence.
  These assets can be computed on-line, but we include in the source code a script to configure and pre-compute the noise effects, reducing the on-line computational load to a simple texture lookup. In order to produce animations that can loop seamlessly, the textures are generated by moving a 2D plane along a circular path through 4D noise, in such a way that the position of the plane for the last recorded frame lines up with its initial position.
- The last option for gas visualization included in the simulator uses a method to perform volumetric rendering through ray marching [39,40].
  For every pixel in the screen, a ray is sent forwards and a number of gas concentration samples are taken along the ray to calculate the optical depth along the ray. This value is then used to compute the transmittance along the ray, simulating the absorption and scattering of light (Figure 6).

Beer–Lambert’s Law [41] is used to compute the transmittance through the gas cloud as a function of the optical depth along the ray, the molar absorptivity of the gas or aerosol (α), and its scattering coefficient (β):

(1) $$\tau \left(x,x^{\prime }\right)=e^{-d_{x,x^{\prime }}\cdot \left(\alpha +\beta \right)}$$

In this expression, *x* and $x^{\prime }$ denote the origin and end of the ray, and $d_{x,x^{\prime }}$ is the optical depth along the ray, calculated by sampling the gas concentration distribution that models the filament $d=\sum_{p}Concentration\left(p\right)\cdot l$. The resulting transmittance value is then multiplied by the color of the background to simulate light being attenuated when travelling through the cloud. The gas concentration is expressed in mol·L${}^{-1}$, and the distance between sampling points (l) in cm, therefore the optical depth is expressed in mol·L${}^{-1}\cdot$cm. The sum of α and β, often referred to as the molar attenuation coefficient, has the units L·mol${}^{-1}\cdot$cm${}^{-1}$.

Simulating the effect of lighting on the gas plume requires an extra step for every sampling point. For a point *p* along the ray where a gas concentration sample is taken, it must be calculated how much light reaches *p* from the light source position *s*, how much of that light is scattered towards the camera, and what proportion of that scattered light actually reaches the camera:

(2) $$Light\left(x\right)=Light\left(s\right)\cdot \;\sum_{p\in samples}\tau \left(p,s\right)\cdot Concentration\left(p\right)\cdot l\cdot \beta \cdot \tau \left(x,p\right)$$

The final color for the pixel is thus:

(3) $$Color\_pixel\left(x\right)=\tau \left(x,x^{\prime }\right)\cdot Color\left(x^{\prime }\right)+Light\left(x\right)$$

Here, *x* is the position of the pixel in world coordinates and $x^{\prime }$ is the point where the view ray intersects the objects in the scene.

This method has a significant computational complexity because of the high number of gas concentration samples needed to render the image of the plume. The number of samples that must be taken in total is N·M, where *N* is the number of marching steps along the main view ray and *M* is the number of steps along the rays cast towards the light source. In each sampling position, it is necessary to iterate over the entire list of *F* gas filaments to check which ones are close enough to contribute gas concentration to the sampled point. Thus, the number of operations necessary to calculate the color of each pixel is O(N·M·F).

Despite this high computational load, it is possible to run it in real time (https://youtu.be/MN9QuFyRQt0 (accessed on 13 March 2021)), since the main ray marching functionality is highly parallelizable, and has been implemented to be run on the GPU as a Compute Shader, in such a way each ray is handled by a separate thread.

Still, the computational load grows quickly as the simulation advances and a higher number of filaments is present in the scenario at once, which can make the rendering too demanding for real-time applications. For that reason, we have introduced some extra optimizations, such as:

- Dividing the space in a grid of cells and assigning to each cell a list of the filaments it contains [42]. This way, when sampling the gas concentration at a given point, it is only necessary to iterate over that smaller list, as opposed to over the list of all filaments. This spatial division is done on the CPU, and can be done in parallel to the GPU rendering, using the time during which the CPU would be idle waiting for the GPU to finish the calculations of the previous step.
- Allowing the ray marching to finish early if the transmittance goes below a certain threshold, since at that point extra steps will not change the visual appearance of the plume. This can be done on both the main view ray, and the secondary rays cast towards the light source.

It should be noted that these optimizations, despite having a noticeable effect on the computation time, do not affect the asymptotic complexity, as the worst-case scenario—when all filaments are in the same space subdivision and the transmittance does not fall below the established threshold—is still O(N·M·F).

### 3.4. ROS Interaction and Robotic Control

The Unity engine itself offers many tools for the creation and management of autonomous agents, which may be used to exploit these simulated environments, but on-line communication with other ROS tools to handle the simulation of mobile robotics is also possible.

The ROS package Rosbridge [43] offers communication between any external application and ROS by exchanging JSON messages through web sockets, making it possible for the external program—in our case, a Unity C# script—to access any ROS functionality (subscribe or publish to topics, communicate with action servers, etc.).

On the Unity side, the library ROSUnity [44] implements many examples of communication through rosbridge, as well as a class structure that can easily be extended to meet the requirements of a new application. Other works that use this project to perform communication between Unity and ROS have already been published [30,45].

## 4. Use Cases

In this section, we discuss a series of applications that benefit of the proposed simulation framework, simplifying their testing and analysis.

### 4.1. Gas/Volatile Source Localization with Semantics

Previous works have proposed the exploitation of ontologies to tackle the problem of Gas Source Localization [46]. The advantage of this strategy over traditional, olfaction-only algorithms is the possibility to introduce pre-existing knowledge to guide the search (e.g., knowing what types of objects can emit the specific type of gas that is being measured).

Given the fact that information obtained purely through gas sensing is limited due to the characteristics of the sensors (response and recovery times, single-point measurements, etc.), this extra knowledge can be of great aid in directing the navigation of the robot to areas of interest in a much more effective way than plume-tracking or similar strategies (e.g., if the sensors detect carbon monoxide, it is not necessary to track the gas plume step by step; the robot can directly move to the kitchen and check the burner). Furthermore, the identification of objects that are known to be potential emission sources for the measured gas offers a powerful tool for tackling the otherwise very complex problem of source declaration.

Despite these advantages, the development and testing of semantics-based techniques poses important challenges. In [46], authors test the algorithm with a number of simulated experiments, and a real-world validation. The simulated experiments, which due to their reproducibility and ease of setup are more extensive than the real ones, were carried out using GADEN alone. Because of this, the simulation did not include visual sensing, and the part of the search that corresponds to object recognition had to be assumed to have been carried out off-line prior to the search itself. Even though this assumption is acceptable for some applications, in which the environment where the search is to take place is known in advance, it still limited the environmental configurations that could be tested (e.g., dynamically changing environments, finding unknown objects at runtime), thus making the simulated experiments less effective at representing the actual performance of the algorithm.

**Figure 7:**
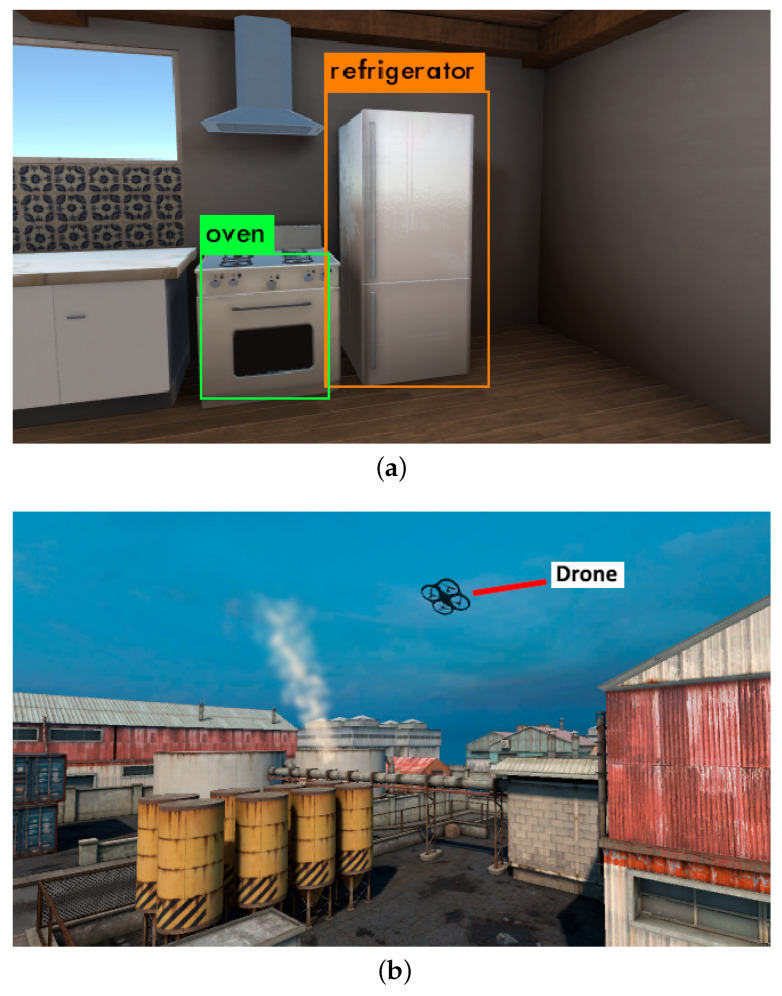
Examples of simulations carried out in with the proposed framework, as represented in the Unity engine. (**a**) The high degree of visual fidelity of the environments is specially relevant for semantics-based applications. In this example, the YOLO neural network [47] is able to recognize relevant items that could be sources of certain gas emissions. (**b**) Simulating the visuals of the gas plume is key for testing methods where the robots rely on vision to locate a gas release.

Using the framework that we propose in this work, it is possible to run both parts of such algorithms (olfactory sensing and object recognition) simultaneously, thus allowing for a more realistic simulation of the enviromental situations the robot might encounter during an actual search (Figure 7a). This makes it possible to obtain more accurate results about the performance of the algorithm than would be possible with the previous, simplified simulated experiments, while maintaining the reproducibility and quick set-up that made simulations more convenient than real experimentation.

### 4.2. Pipe Leak Detection with Plume Imaging

Numerous techniques have been proposed to detect and track gas plumes through computer vision [21,48,49]. The tracking of a gas plume with a camera, whether on its own or complementing an olfactory sensor, offers interesting advantages. Namely, it makes it possible to observe the gas plume from far away before it can be detected by gas sensors (Figure 7b), and it allows for the tracking of the plume in a more efficient manner by observing its shape and movement instantaneously, rather than trying to infer them from sparse gas concentration and wind measurements.

For the reasons previously discussed (repeatability of the experiments, ease of setup, etc.), a simulation environment such as the one we propose, which delivers an accurate simulation of the dispersion of the gas and offers tools for a realistic visual representation of the plume, is an important asset for the development and testing of these techniques.

An important limitation of the simulator when applied to these methods is that it does not currently include a way to properly simulate the use of an infrared or ultraviolet cameras. Since many of the gases that need to be monitored or controlled are not observable in the visible spectrum (e.g., $CH_{4}$ is observed in the infrared spectrum, while $SO_{2}$ is tracked with UV cameras), this issue limits the range of substances that can be appropriately simulated. This is an interesting direction for any future work that aims to expand the capabilities of the simulation framework.

## 5. Conclusions and Future Work

In this work, we have presented a new framework for the integration of simulated olfaction and simulated vision. We rely on GADEN to carry out the simulation of gas dispersion, and use the Unity Engine to simulate graphically complex environments and camera vision. By combining these tools, we achieve a level of realism in the simulation that would not be possible with the existing, state-of-the-art alternatives.

We believe our proposal to be a useful asset in the development of multi-sensor olfactory techniques (source localization, distribution mapping, etc.), which are an interesting direction for future research, given that they provide a way to make up for the sparsity of olfactory measurements with other sensory information.

Several directions exist for future work: first, the implementation of new functionalities—such as the implementation of simulated infrared cameras—to broaden the spectrum of applications for which this simulation framework is useful; second, the optimization of the computational aspects of the simulation, which can still be demanding for the real-time rendering of the gas plume; lastly, the usage of the simulation framework to develop and test specific robotics applications that exploit the characteristics it provides.

## Figures and Tables

**Table 1:** Comparison of the features offered by the different alternatives for gas dispersion simulation. Adapted from [17].

		PlumeSim	GADEN	CFD
Environment	Dimensions	2D	3D	3D
	Obstacles	No	Yes	Yes
Gas dispersion	Model	Gaussian/meandering plume	Filament model	Numerical
	Number of sources	1	Multiple	Multiple
Integration	Sensors	MOX	PID & MOX	No
	Robotics platform	ROS pkg	ROS pkg	No
